# High expression of KITLG is a new hallmark activating the MAPK pathway in type A and AB thymoma

**DOI:** 10.1111/1759-7714.13486

**Published:** 2020-05-28

**Authors:** Zhaoyu Yang, Shinan Liu, Yuanguo Wang, Yuan Chen, Peng Zhang, Yimei Liu, Hui Zhang, Peng Zhang, Ziyou Tao, Kai Xiong

**Affiliations:** ^1^ Department of Cardiothoracic Surgery Tianjin Medical University General Hospital Tianjin China

**Keywords:** Biomarker, KITLG, MAPK, Thy0517, thymoma

## Abstract

**Background:**

KIT proto‐oncogene ligand (KITLG) is a pleiotropic factor which is found in diverse cancers and is involved in cell proliferation, differentiation, and survival. However, the value of KITLG in thymoma remains unclear.

**Methods:**

A total of 121 thymoma samples from The Cancer Genome Atlas Thymoma (TCGA‐THYM) dataset were used to analyze KITLG related genome‐wide expression profiles, and microRNA profiles and methylation alterations and a GEO dataset‐GSE29695, including 37 samples was used as verification. For cell‐based studies, specific small interfering RNA targeting KITLG or a KITLG overexpression vector were used to clarify the changes of the MAPK pathway in an AB thymoma cell line Thy0517.

**Results:**

Both datasets showed that high expression of KITLG was significantly associated with type A and AB thymoma. Through multiomic analysis of the TCGA‐THYM, it was found that with the high expression of KITLG, there were 220 upregulated and 72 downregulated genes at the mRNA level, 79 positive and 78 negative miRNAs, 28 hypermethylation and 163 hypomethylation regions. In the thymoma cell line Thy0517, it was found that the expression of GRB2 and the phosphorylation levels of BRAF, MEK1/2, and ERK1/2 in the MAPK pathway were positively correlated with the change in KITLG.

**Conclusions:**

High expression of KITLG is a new hallmark of WHO type A and AB thymomas in which it might play a critical role through the activation of the MAPK signaling pathway. Additionally, it is hoped that KITLG will become a potential target for the diagnosis of type A and AB thymoma through further research in the future.

**Key points:**

**Significant findings of the study:**

KIT proto‐oncogene ligand (KITLG) is a new hallmark of type A and AB thymomas which induce a series of aberrant alteration of mRNA, miRNA and DNA methylation. The expression of KITLG is significantly higher in type A and AB than other subtypes of thymoma.

**What this study adds:**

KITLG activated the MAPK signaling pathway to promote type A and AB thymoma which might be a potential diagnostic biomarker or target.

## Introduction

Thymoma is the most common anterior mediastinal tumor with a complex histological classification.^1^ According to the World Health Organization (WHO) classification of this cancer, the pathology of thymoma is divided into five subtypes based on lymphocytic infiltration and the morphology of epithelial cells.^2^ There are many differences between each thymoma subtype from clinical manifestations to gene expression levels. Although surgery is fast and effective for patients with thymoma, 10%–29% of postoperative patients will relapse.^3^ Therefore, effective biomarkers for each type of thymoma for its diagnosis, treatment, and prognosis are urgently required. The biomarkers include a wide variety of mRNA, microRNA (miRNA), non‐coding RNA, and epigenetic factors, some of which have been independently identified.^4^

KIT proto‐oncogene ligand (KITLG), also known as stem cell factor (SCF), is a ligand of the c‐KIT tyrosine kinase receptor with multiple biological functions.^5^ Aggressive expression of KITLG by autocrine/paracrine stimulation‐circulatory mechanisms is required for tumors, and it has been identified in multiple cancer types such as glioma, non‐small cell lung cancer (NSCLC), and colorectal cancer.^6^ The MAPK pathway as a classical signaling pathway has an important role in regulating cell proliferation, differentiation, migration, and survival.^7^ A previous study showed that this pathway is the core process behind cellular activity in chronic lymphocytic leukemia.^8^ One of its upstream factors, KITLG, is highly expressed, indicating a link between KITLG and this pathway. However, to date, the diagnostic and prognostic value of KITLG and its downstream signaling pathways in thymoma remain unclear.

In this study, we used bioinformatics methods to search KITLG‐related genomics and methylation data for thymoma in TCGA and GEO databases. In addition, we used the AB type thymoma cell line Thy0517^9^ to further explore KITLG and the changes of MAPK pathway in cell experiments. Our work aimed to offer more related evidences for KITLG expression as a potential biomarker for patients with type A and AB thymoma.

## Methods

### Samples and genomics profile datasets

We obtained RNA‐seq gene expression version 2 (RNA Seq V2) level 3 data (Data Release 9.0, Release Date: October 24, 2017) from post‐surgery thymoma tissue samples from thymoma patients of each WHO type from the UCSC XENA website (http://xena.ucsc.edu/public). We performed the analysis of RNA‐seq data of 121 thymoma samples. In addition, another independent cohort (GEO accession number: GSE29695) including 37 patients with thymic neoplasms from Gene Expression Omnibus (GEO) database was used as verification.^10^

### RNA‐seq data analysis

In a recent study, due to the availability of large sets of various subtype thymoma samples, and fewer adjacent normal datasets, analyses of differentially expressed genes in GEO data were conducted by applying a new method in which the differentially expressed genes are identified by comparing subtype A and AB thymoma samples with type B1, B2, B3 and C thymoma.^11^

**Figure 1:**
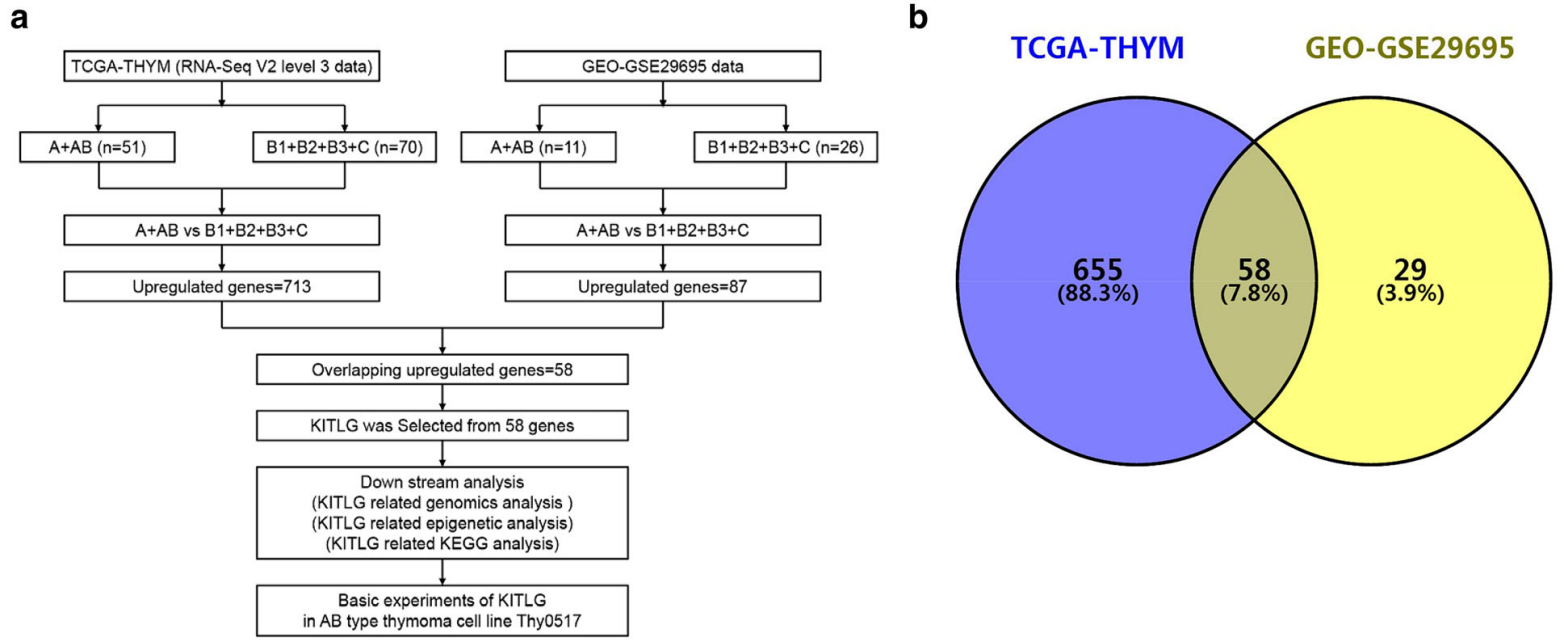
Differentially expressed genes analyses, using publicly available online bioinformatics databases. (**a**) Overview of analysis and experiments about KITLG from TCGA‐THYM and GSE29695 datasets. (**b**) Venn diagram of overlapping upregulated genes in type A + AB thymoma (vs. type B1 + B2 + B3 + C thymoma) from TCGA‐THYM and GEO‐GSE29695 datasets.

In this study, we also applied such an approach. We segregated TCGA‐THYM tumor samples into two groups: 51 A and AB subtypes (A + AB), as well as 70 B1, B2, B3, and C subtypes (B1 + B2 + B3 + C). The samples from GEO‐GSE29695 were also divided into two groups: 11 A + AB subtypes, as well as 26 B1 + B2 + B3 + C subtypes. First, we carried out an analysis of differentially expressed genes in these two groups of TCGA and GEO using R/Bioconductor package and GEO2R (https://www.ncbi.nlm.nih.gov/geo/geo2r/). Upregulated genes were identified separately by applying a Benjamini‐Hochberg (BH) false‐discovery rate (FDR) of *P* < 0.01 with a log‐fold change (logFC) >1.5. Finally, we obtained the overlapping upregulated genes from both datasets using “Venny 2.1” (https://bioinfogp.cnb.csic.es/tools/venny/index.html) for further analysis. KITLG was selected from 58 overlapping upregulated genes after analyzing the significance of differential genes and the relationship with tumor growth. Additionally, the median value of KITLG expression was used to divide 121 patients into high expression of KITLG (KITLG^high^) and low expression of KITLG (KITLG^low^) groups. We first sorted out the expression values of KITLG of 121 thymoma samples in the TCGA‐THYM data set, and then found the median of all these values. There were 61 samples in which expression of KITLG were greater than or equal to the median, classified as the group KITLGhigh, while another 60 samples with KITLG expression less than the median were classified as the group KITLGlow. KITLG‐related genomic, epigenetic and KEGG analyses were based on these two groups from TCGA‐THYM data. All experimental design, quality control, and data normalization were in line with the standard protocols. Finally, we conducted basic investigations into KITLG using the type AB thymoma cell line Thy0517 (Fig 1).

### Cell culture

The human thymoma cell line Thy0517 (PMID: 26273358, DOI: 10.1111/1759‐7714.121 63) was established from thymoma type AB patients by tissue explant and cultured by the Department of Cardiothoracic Surgery, Tianjin Medical University General Hospital. We own the patent of this cell line. Cells were cultured in DMEM (Gibco) supplemented with 10% fetal bovine serum (Clark Bioscience), 100 U/mL penicillin, 100 μg/mL streptomycin (Sigma‐Aldrich), and 2 μg/mL amphotericin (Sigma‐Aldrich) at 37°C in the presence of 5% CO_2_.

### KITLG small‐interfering RNA (siRNA) silencing

KITLG‐siRNA were purchased from GenePharma (Shanghai, China). The oligonucleotide sequences were as follows: 5′‐GCGAGAUGGUAGUACA AUUTT‐3′ for the sense strand and 5′‐AAUUGUACUACCAUCUCGCTT‐3′ for the antisense strand. A negative control siRNA was used in parallel. Thy0517 cells were transfected with 25 μM siRNA against KITLG using Lipofectamine 3000 (Invitrogen) according to the manufacturer's instructions. The cells were cultured in Opti‐MEM (Gibco) at 37°C in the presence of 5% CO2 for 6 hours. Total RNA and total protein were prepared 48 hours post‐transfection and used for real‐time PCR and western blot analysis, respectively.

### KITLG‐overexpressing plasmid (pcDNA3.1(+)‐KITLG) construction

pcDNA3.1(+)‐KITLG was obtained from GeneChem (Shanghai, China). Thy0517 was transfected with pcDNA3.1(+)‐KITLG to induce overexpression of KITLG and with the empty pcDNA3.1(+) vector as a control. Transfections were performed using Lipofectamine 3000 according to the manufacturer's instructions.

### RNA extraction and real‐time PCR

Total RNA was extracted using TRIzol (TaKaRa) according to the manufacturer's instructions, and reverse‐transcribed using a reverse transcriptases M‐MLV kit (TaKaRa). qPCR was performed in a real‐time PCR system (Applied Biosystems) using TB Green Premix Ex Taq II (TaKaRa). Cycling conditions were 95°C for 2 minutes, followed by 40 cycles at 95°C for 30 seconds, 56°C for 30 seconds, and 72°C for 30 seconds. The primer sequences are shown in Table 1.

**Table 1:** Primer sequences for real‐time PCR

Primers	Sequences
KITLG ‐ Forward	5′‐ AGCCTTATACTGGAAGAAGAGAC ‐ 3′
KITLG ‐ Reverse	5′‐ GTTGATACAAGCCACAATTACAC ‐ 3′
GRB2 ‐ Forward	5′‐ TTCTCCCTCTCTGTCAAGTTTG ‐ 3′
GRB2 ‐ Reverse	5′‐ GTATGTCGGCTGCTGTGG ‐ 3′
BRAF ‐ Forward	5′‐ ATGCTGTGGTTCTTGTATCTTG ‐ 3′
BRAF ‐ Reverse	5′‐ GACTCCGCTCTCCTCTGG ‐ 3′
MEK1 ‐ Forward	5′‐ GAAGGAGATGCGGCTGAG ‐ 3′
MEK1 ‐ Reverse	5′‐ GAGGAGGCTCGTTGACTATG ‐ 3′
MEK2 ‐ Forward	5′‐ TCCTGGACTATATTGTGAACGAG ‐ 3′
MEK2 ‐ Reverse	5′‐ GTGTGGTTTGTGAGCATCTTC ‐ 3′
ERK1 ‐ Forward	5′‐ GACGGAGTATGTGGCTACG ‐ 3′
ERK1 ‐ Reverse	5′‐ CCAGGATGCCCAGAATGTG ‐ 3′
ERK2 ‐ Forward	5′‐ CCGAGTGACGAGCCCATC ‐ 3′
ERK2 ‐ Reverse	5′‐ GTCCTCTGAGCCCTTGTCC ‐ 3′
GAPDH ‐ Forward	5′‐GCTCTCTGCTCCTCCTGTTC‐ 3′
GAPDH ‐ Reverse	5′‐CGACCAAATCCGTTGACTCC‐ 3′

KITLG, KIT ligand; GRB2, growth factor receptor‐bound protein 2; BRAF, serine/threonine‐protein kinase B‐raf; MEK1/2, dual specificity mitogen‐activated protein kinase kinase 1/2; ERK1, mitogen‐activated protein kinase 3; ERK2, mitogen‐activated protein kinase 1; GAPDH, glyceraldehyde‐3‐phosphate dehydrogenase.

The reactions were conducted three times, and threshold cycle values were normalized to the expression of GAPDH mRNA. The specificity of the products was determined by melting curve analysis. Relative mRNA expression of the target genes was obtained by normalization to the control group and to GAPDH levels with the 2^‐△△Ct^ method.

### Western blot analysis

The harvested cells were suspended in cell lysis buffer (Beyotime). After 12% SDS‐PAGE electrophoresis, the proteins were transferred onto nitrocellulose membranes (Amersham), and blocked with 5% skim milk prior to primary antibody incubation overnight at 4°C. After washing, the membranes were incubated with HRP‐conjugated secondary antibodies (Proteintech, 1:4000) for 1 hour. The proteins were detected using ECL reagent (GE Healthcare Life Sciences) and images were captured by a chemiluminescent imaging system (Tanon). The list of antibodies are shown in Table 2.

**Table 2:** Primary antibodies for western blot analysis

Antibody	Manufacturer	Product #	Source	Type	Dilution
KITLG	Proteintech	26 582‐1‐AP	Rabbit	Polyclonal	1:800
GRB2	CST	3239	Rabbit	Monoclonal	1:1000
BRAF	Proteintech	20 899‐1‐AP	Rabbit	Polyclonal	1:800
phospho‐BRAF	CST	2696	Rabbit	Polyclonal	1:1000
MEK1/2	CST	8727	Rabbit	Monoclonal	1:1000
phospho‐MEK1/2	CST	9154	Rabbit	Monoclonal	1:1000
ERK1/2	CST	4695	Rabbit	Monoclonal	1:1000
phospho‐ ERK1/2	CST	4370	Rabbit	Monoclonal	1:2000
GAPDH	Proteintech	60 004‐1‐Ig	Mouse	Monoclonal	1:5000

KITLG, KIT ligand; GRB2, growth factor receptor‐bound protein 2; BRAF, serine/threonine‐protein kinase B‐raf; MEK1/2, dual specificity mitogen‐activated protein kinase kinase 1/2; ERK1, mitogen‐activated protein kinase 3; ERK2, mitogen‐activated protein kinase 1; GAPDH, glyceraldehyde‐3‐phosphate dehydrogenase.

### Statistical analysis

Student's *t*‐test and multiple hypothesis correction were used to identify genome‐wide differences in gene, miRNA, and methylation profiles between the KITLG^high^ and KITLG^low^ groups. All analyses were performed using the R limma software package. qPCR and protein data were depicted as the mean ± SEM. Comparisons between groups were evaluated by one‐way ANOVA test using GraphPad Prism 7.0 software. Statistical significance was considered when *P* < 0.05.

## Results

### KITLG overexpression in type A and AB thymoma

**Figure 2:**
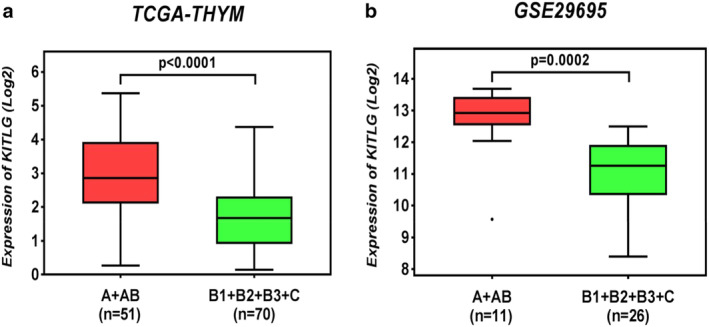
The expression of KITLG in thymoma. (**a**) TCGA‐THYM dataset: A + AB (*n* = 51) versus B1 + B2 + B3 + C (*n* = 70). (**b**) GEO‐GSE29695: A + AB (*n* = 11) versus B1 + B2 + B3 + C (*n* = 26).

Two public microarray datasets were used to compare KITLG expression between A + AB with B1 + B2 + B3 + C thymoma patients. KITLG was highly expressed in type A and AB thymoma (51 A + AB s 70 B1 + B2 + B3 + C, TCGA‐THYM; *P* < 0.001; Fig 2a), which was validated in the GEO dataset (11 A + AB vs. 26 B1 + B2 + B3 + C, GSE29695; *P* < 0.001; Fig 2b). The two datasets showed the same trend of significant overexpression of KITLG in type A and AB thymoma compared with other subtypes.

### KITLG‐related genome‐wide expression profiles

**Figure 3:**
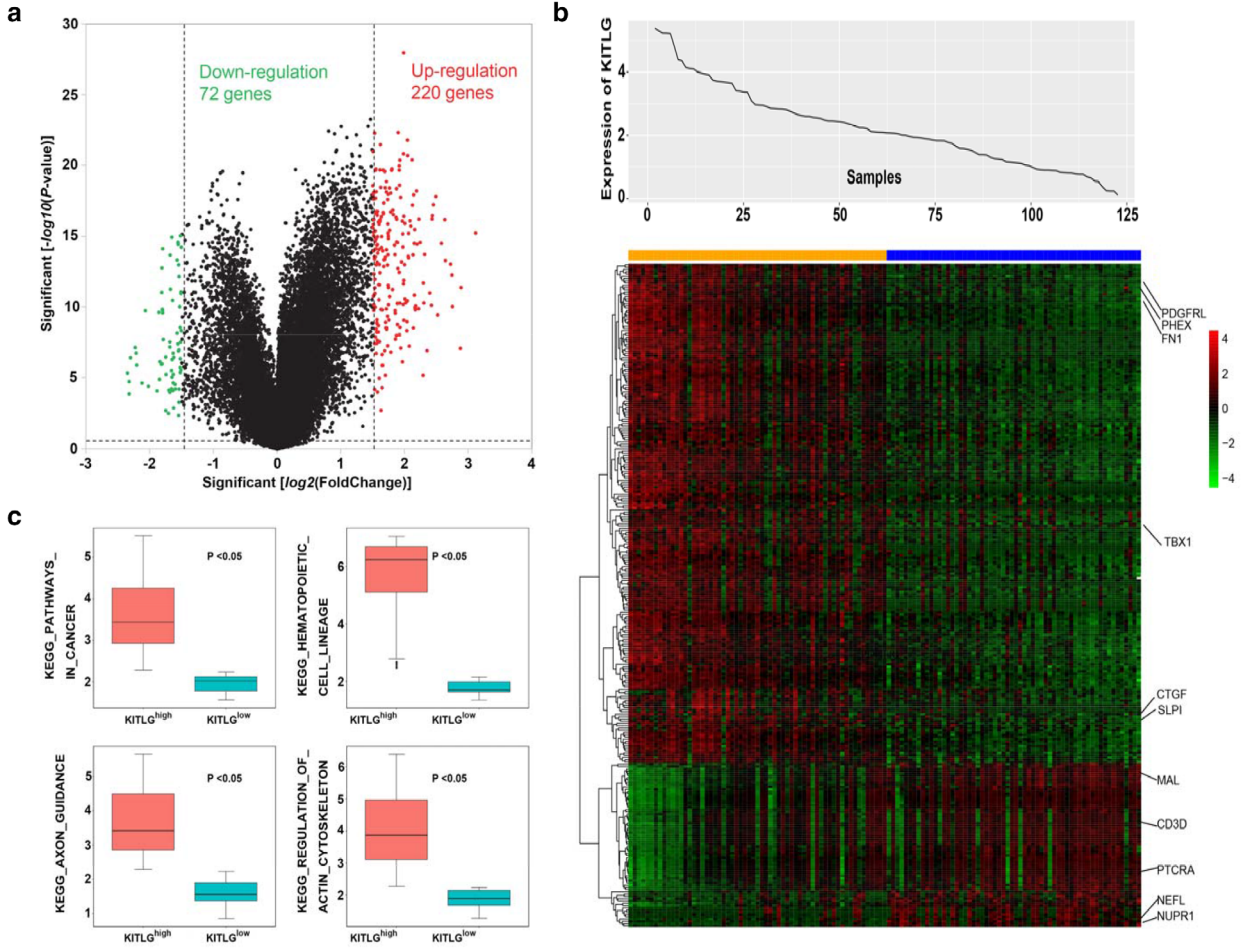
KITLG related genome‐wide expression profiles. (**a**) Volcano plot of differential genes expression between KITLG^high^ and KITLG^low^. (**b**) Heatmap of genes associated with KITLG. The top curve shows KITLG's expression distribution of 121 thymoma samples (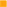) KITLG (high) (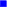) KITLG (low). (**c**) Boxplots of KITLG‐related upregulated signaling pathways.

To further investigate the biological role of KITLG in thymoma, gene expression profiles associated with KITLG were derived based on genome‐wide microarray analysis. A total of 220 upregulated and 72 downregulated genes (*P* < 0.01 and log FC > 1.5 or log FC < ‐1.5) were identified as being significantly associated with KITLG expression (Fig 3a). As for upregulated genes, platelet‐derived growth factor receptor (PDGFR)^12^ and connective tissue growth factor (CTGF)^13^ were associated with thymic tumor risk and survival. The levels of fibronectin (FN1)^14^ and T‐box transcription factor 1 (TBX1),^15^ which are related to thymus development, became high. As for downregulated genes, PTCRA,^16^ which is involved in negative regulation of thymic epithelial cell proliferation and differentiation, showed low levels. The decreased expression of CD3D^17^ and T cell differentiation protein (MAL)^18^ might be associated with autoimmunity, which could provide some reasons as to why thymoma is always accompanied by autoimmune disease. The differentially expressed genes are presented with KITLG expression in a heatmap (Fig 3b).

Furthermore, the relationship between KITLG expression and cell signaling pathways was analyzed using the Molecular Signatures Database (MSigDB, v7.0).^19^ The expression levels of signaling pathways were quantified by counting the mean expression of the whole genes within it and dividing them into different groups according to the expression of KITLG. Finally, four upregulated pathways were found to be significantly associated with KITLG^high^ (*P* < 0.05). Pathways involved growth, proliferation, and differentiation of thymoma were upregulated, including pathways in cancer and pathway of hematopoietic cell lineage. Other molecule‐related pathways, such as axon guidance and regulation of actin cytoskeleton were also expressed at higher levels (Fig 3c).

### KITLG‐related miRNA profiles

**Figure 4:**
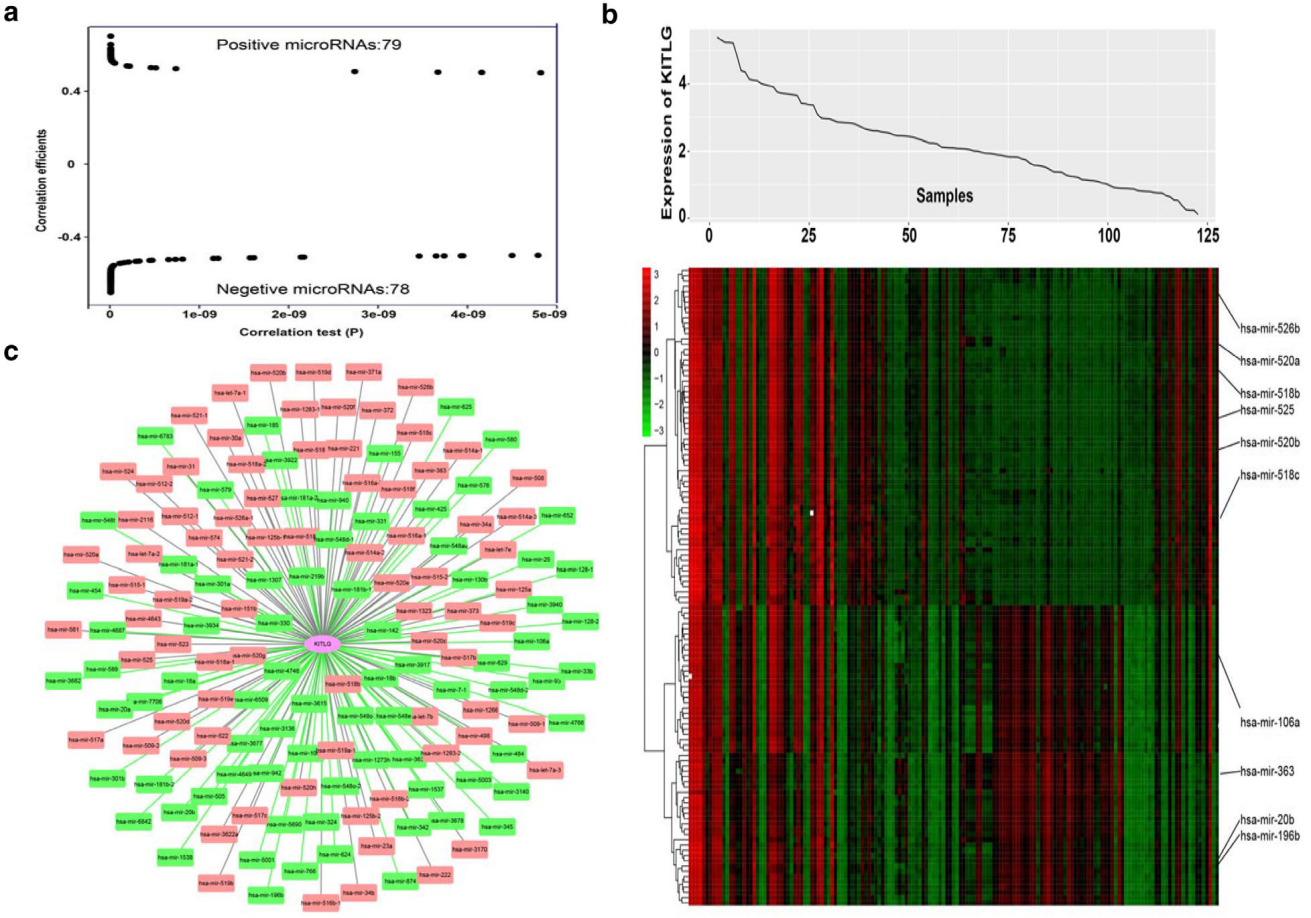
KITLG related genome‐wide microRNAs profiles. (**a**) Volcano plot of positive and negative microRNAs. (**b**) Heatmap of microRNAs associated with KITLG. The top curve shows KITLGs expression distribution of 121 thymoma samples. (**c**) Interaction network of differentially expressed microRNAs and KITLG.

miRNA data was excavated from TCGA‐THYM to further explore the correlation with KITLG expression. A total of 79 positive and 78 negative miRNAs were identified (*P* < 0.05, Pearson R > 0.5 or < −0.5, Fig 4a). As previous studies reported, some of these miRNAs were closely associated with thymoma or the thymus. As for upregulated miRNA, miR‐125a regulates FOXP3 expression and modulates the different inflammatory signaling pathways; this might be associated with thymoma with myasthenia gravis.^20^ High expression of miR‐34a might modulate thymoma cell differentiation and development.^21^ As for downregulated miRNA, miR‐106 can target MEK2, which might affect the thymus' immune function.^22^ Meanwhile, MEK2 is a member of the MAPK signaling pathway, which was also upregulated in type A and AB thymoma, as previously mentioned. miR‐363 could regulate TNF receptor superfamily member 5 and cyclin‐dependent kinase inhibitor 1A, which might influence the tumor cell cycle.^23^ Low levels of miR‐20b expression were also evident in thymoma. MiR‐20b acts as a tumor suppressor in the development of thymoma by repression of NFAT5 and CAMTA1 expression to inhibit NFAT signaling.^24^ MiR‐7 can control CCL21 release, which is essential for thymoma germinal center development.^25^ The differentially expressed miRNAs are also presented with KITLG expression in a heatmap (Fig 4b). An interaction network of differentially expressed miRNAs and KITLG was established using Cytoscape 3.7.1^26^ to further demonstrate their relationship (Fig 4c). KITLG as the core was at the center of the network. The upregulated miRNAs are represented by pink rounded rectangles, while the downregulated miRNAs are labeled by green rounded rectangles.

### Upregulated expression of KITLG related methylation alterations

**Figure 5:**
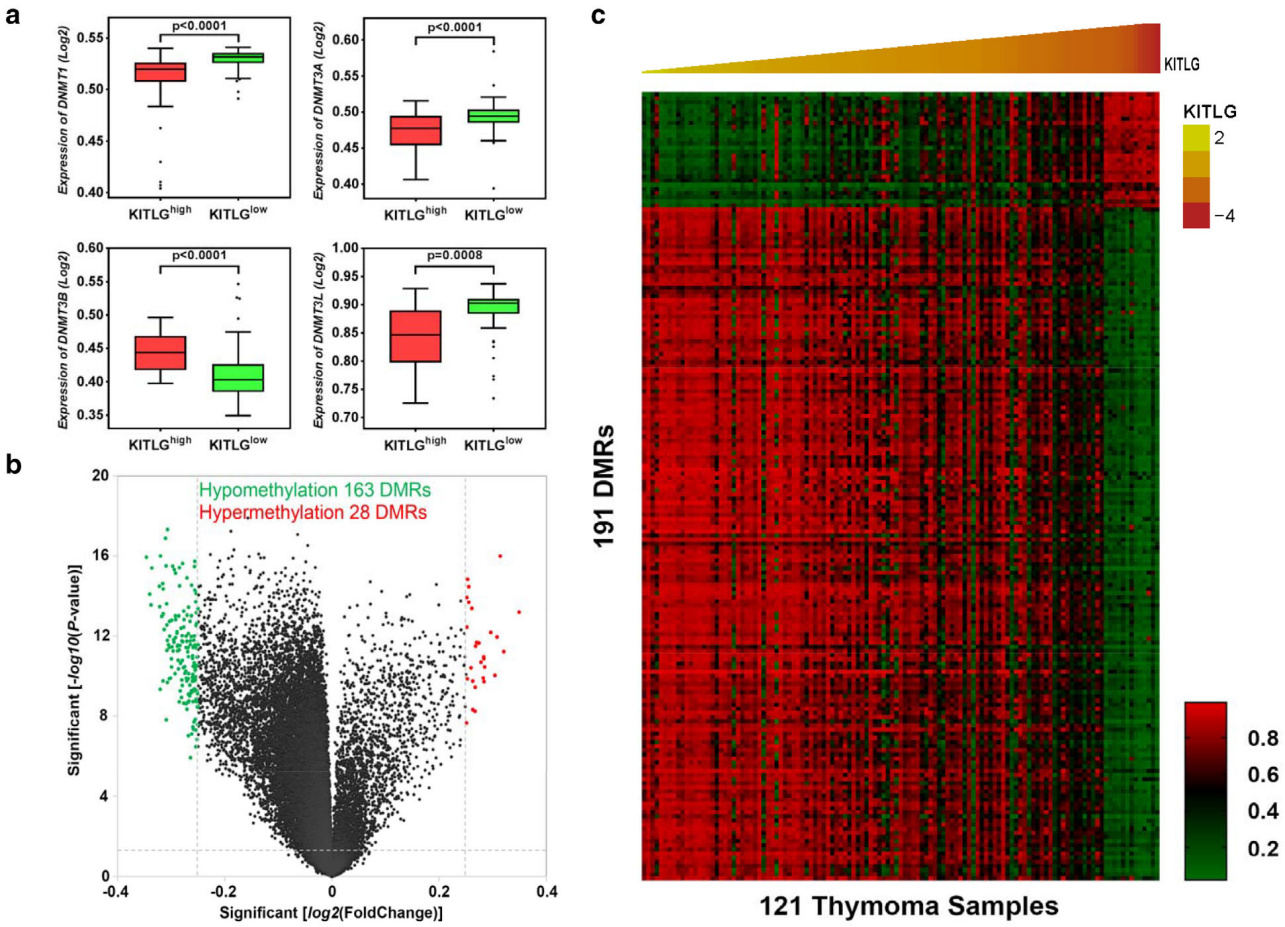
Elevated expression of KITLG corresponding genome‐wide methylation alterations. (**a**) The expression of DNA methyltransferases (DNMT1, DNMT3A, DNMT3B, DNMT3L) in groups KITLG^high^ and KITLG^low^. (**b**) Volcano plot of hypermethylation and hypomethylation DMRs. (**c**) Methylation heatmap of 191 DMRs associated with KITLG.

The development of thymoma is a multistep process involving epigenetic alterations. Increasing numbers of reports have provided evidence that aberrant DNA methylation, especially that by DNA methyltransferases, has a critical role in the different subtypes of thymoma.^27^ DNMT1, together with DNMT3A, DNMT3B, and DNMT3L cooperating with each other, are a group of catalytically inactive DNA methyltransferases.^28^ Compared with the KITLG^high^ and KITLG^low^ groups, using TCGA‐THYM data, DNMT3B showed significantly higher expression in the KITLG^high^ group (*P* < 0.0001). The level of DNMT1, DNMT3A, and DNMT3L appeared lower in the KITLG^high^ group (*P* < 0.001), which was different from other tumors (Fig 5a). Next, there were 28 hypermethylation and 163 hypomethylation differential methylated regions (DMRs) that differed between the KITLG^high^ and KITLG^low^ groups (*P* < 0.05, LogFC >0.25 or < −0.25; Fig 5b). Finally, the 191 DMRs were presented with KITLG as a heatmap (Fig 5c).

### KITLG‐siRNA inhibits the GRB2/BRAF/MEK/ERK pathway in type AB thymoma cells

A previous study of chronic lymphocytic leukemia demonstrated that KITLG is an upstream moderator of the MAPK pathway.^8^ From the bioinformatics analysis of TCGA‐THYM and GEO‐GES29695 data, we found that the expression of KITLG was higher in type A and AB thymoma than in the other subtypes. Moreover, many genes and signaling pathways related to KITLG, including the MAPK pathway, were also more positively expressed in type A and AB thymoma. To identify the significance of KITLG with the MAPK pathway in thymoma, we performed an in vitro experiment using the human thymoma AB cell line Thy0517, which has high expression of KITLG.

**Figure 6:**
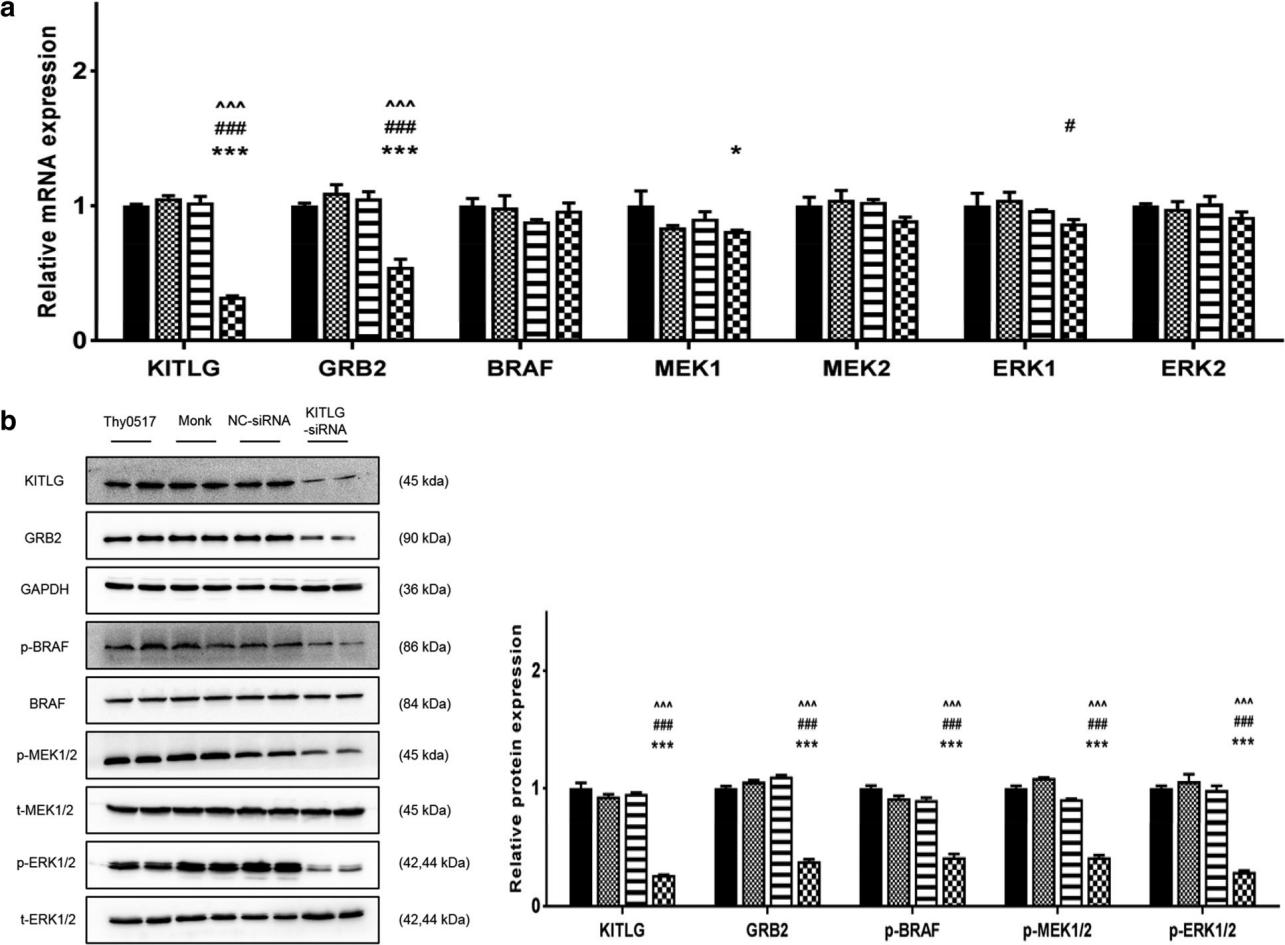
Downregulated KITLG inhibited MAPK signaling pathway in Thy0517 cells. The Thy0517 cell line was transfected with KITLG‐siRNA, or NC‐siRNA, along with a mock transfected negative control. (**a**) The mRNA levels of KITLG, GRB2, BRAF, MEK1, MEK2, ERK1, ERK2 and GAPDH were determined by rtPCR. (**b**) The protein levels of KITLG, GRB2, p‐BRAF/t‐BRAF, p‐MEK1/2/ t‐MEK1/2, p‐ERK1/2/ t‐ERK1/2 and GAPDH were determined by western blot (a,b (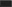) Thy0517 (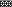) Monk (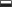) NC‐siRNA (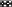) KITLG‐siRNA). All values are expressed as mean ± SEM. *P < 0.05, *** *P* < 0.001 versus Thy0517; # *P* < 0.05, ### *P* < 0.001 versus Monk; ^^^ *P* < 0.001 versus NC‐siRNA.

We set up in vitro cell experiments consisting of a Thy0517 group, a mock group (transfection reagent only), an NC‐siRNA group, and a KITLG‐siRNA group. Thymoma cells were transfected with NC‐siRNA or KITLG‐siRNA.Then, 48 hours post‐transfection, the mRNA and protein levels of KITLG as well as key factors of the MAPK pathway were analyzed. The results showed that both the mRNA and protein levels of KITLG was significantly decreased compared with the control groups (*P* < 0.001) along with a corresponding decrease in GRB2, which is also a critical upstream protein in the MAPK pathway (*P* < 0.001), whereas the mRNA levels of BRAF, MEK1/2, and ERK1/2 remained almost unchanged *(P* > 0.05) (Fig 6). However, the protein levels of p‐BRAF, p‐MEK1/2, and p‐ERK1/2 were also significantly reduced compared with the control groups (*P* < 0.001) (Fig 6b).

### KITLG overexpression plasmid aggravates activation of the MAPK pathway in type AB thymoma cells

**Figure 7:**
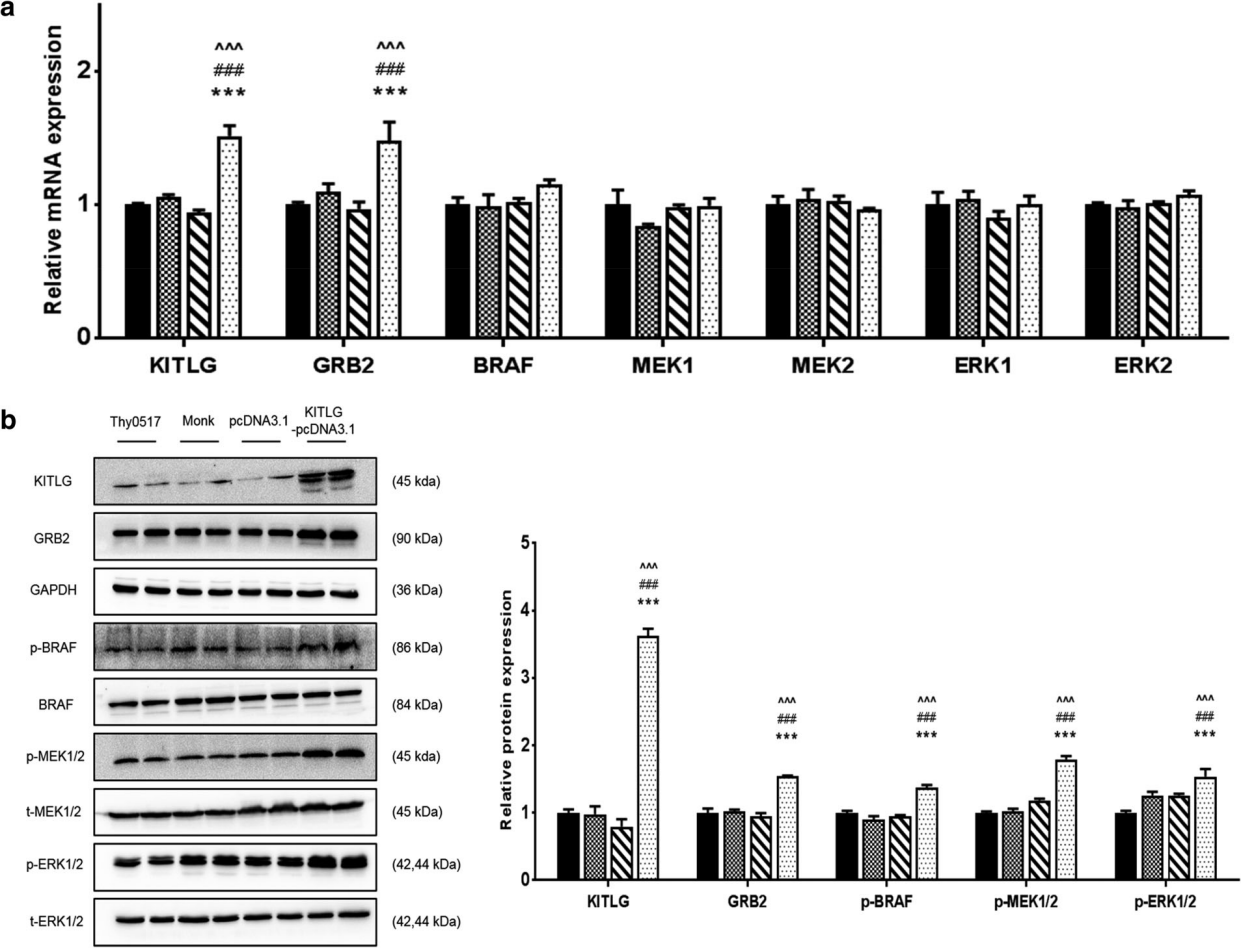
Upregulated KITLG activated MAPK signaling pathway in Thy0517 cells. The Thy0517 cell line was transfected with KITLG‐pcDNA3.1(+), or pcDNA3.1(+), along with a mock transfected negative control. (**a**) The mRNA levels of KITLG, GRB2, BRAF, MEK1, MEK2, ERK1, ERK2 and GAPDH were determined by rtPCR. (**b**) The protein levels of KITLG, GRB2, p‐BRAF/t‐BRAF, p‐MEK1/2/ t‐MEK1/2, p‐ERK1/2/ t‐ERK1/2 and GAPDH were determined by western blot (a,b (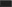) Thy0517 (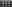) Monk (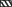) pcDNA3.1(+) (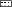) KITLG‐pcDNA3.1(+)). All values are expressed as mean ± SEM. ***P < 0.001 versus Thy0517; ###*P* < 0.001 versus Monk; ^^^*P* < 0.001 versus pcDNA3.1(+).

To provide additional evidence that KITLG is involved in the activation of the MAPK pathway in type AB thymoma cells, KITLG overexpression plasmid was used to enhance its expression in Thy0517 cells. We set up four groups, including a Thy0517 group, a mock group, a pcDNA3.1(+) group, and a pcDNA(+)‐KITLG group. Thy0517 cells transfected with empty pcDNA(+) plasmid or pcDNA(+)‐KITLG were cultured for 48 hours following transfection. The levels of KITLG mRNA and protein were remarkably upregulated in the pcDNA3.1(+)‐KITLG group compared with the negative control groups (*P* < 0.001). As expected, the mRNA and protein levels of GRB2 showed the same trends as KITLG (*P* < 0.001) (Fig 7a,b). In addition, there were no changes in the mRNA levels of BRAF, MEK1/2, and ERK1/2 (*P* > 0.05) (Fig 7a), while the protein expression of p‐BRAF, p‐MEK1/2, and p‐ERK1/2 was notably upregulated by pcDNA3.1(+)‐KITLG plasmid (*P* < 0.001) (Fig 7b).

## Discussion

Herein, we demonstrate that an important gene, KITLG, is a genomic hallmark of type A and AB thymomas. KITLG interacts with its receptor c‐KIT to perform important roles which has been found in various tumors.^29^ However, the mechanism of KITLG overexpression in thymoma is currently unknown. Fortunately, the TCGA network provides valuable information regarding the genetic changes in critical genes involved in the cancer pathways in different subtypes of thymoma patients. Previous studies have reported several molecular signatures that could be used as biological markers in distinguishing thymoma subtypes. The most well‐known is GTF2I, the mutation of which is predominantly observed in type A and AB thymoma.^30^ HRAS and NRAS are also found at high levels in subtype A and AB, while TP53 is predominantly observed in type C thymoma.^31^ However, the current known biomarkers for distinguishing thymoma subtypes are not as yet clearly defined. In this study, we identified another molecule, KITLG, in thymoma. The KITLG^high^ group contained significantly more patients with the A and AB subtypes, suggesting KITLG might be another independent biomarker of type A and AB thymoma.

In addition, mRNA expression, as well as post‐transcriptional regulation by miRNA and DNA methylation, also have critical roles in thymoma genesis. Thus, we analyzed the effects of KITLG overexpression in thymoma from these three aspects. First, we investigated KITLG‐associated gene expression profiles and related cell signaling pathways. Second, we constructed an interaction network of differentially expressed miRNAs along with high expression of KITLG to reveal the complex associations between them. The ceRNA mechanisms could be predicted based on these miRNAs. Finally, we realized that differentially methylated genes related to KITLG^high^ could contribute to the pathogenesis of type A and AB thymoma. DNMT3B, as a DNA methyltransferase, was expressed at notably higher levels in the KITLG^high^ group. We also found hypomethylated DMRs were more apparent in the KITLG^high^ group compared with hypermethylated DMRs, which might result from the decline of tumor suppressors among the KITLG^high^ expressers. This abnormal methylation may provide additional information regarding type A and AB thymoma.

As previous studies reported, KITLG can result in the activation of several downstream signaling pathways, such as STAT and PI3K/AKT. In detail, overexpressed KITLG is released by keratinocyte‐activated melanocyte via the MAPK pathway.^32^ The proliferation and invasion of c‐KIT‐positive colorectal cancer cells was demonstrated to be enhanced by KITLG through the PI3K/AKT signaling pathway.^33^ In this study, we found that MAPK signaling pathway was the major upregulated pathway associated to KITLG in type A and AB thymoma based on TCGA‐THYM data. To confirm this point, we used established AB thymoma cell line Thy0517 and the results showed that KITLG suppression could downregulate the expression of GRB2, p‐BRAF, p‐MEK1/2, and p‐ERK1/2, whereas KITLG overexpression had a profound activating effect on the MAPK pathway in Thy0517.

Thymoma has strong association with autoimmune diseases, especially myasthenia gravis. A previous study reported that KITLG as a critical mast cell growth factor, secreted by Th17 cell‐mediated keratinocytes, could stimulate the accumulation and proliferation of mast cells, and mast cells could act as antigen‐presenting cells or effector cells secreting various immune responses to promote immune disorders.^34^ Combined with our latest research (unpublished data), we found that the balance of Th17/Treg was abnormal and the antigen presentation ability of dendritic cells improved in the thymoma microenvironment, which impacted the positive and negative selection of T cells. It may be that excessive Th17 cells promoted overexpression of KITLG in thymoma, leading to dendritic cells, macrophages, and mast cells being involved in aberrant immune responses. Thus, KITLG is perhaps another area for the exploration of thymoma with autoimmune disease.

With the development of precision medicine, accurate histological diagnosis of thymoma WHO subtypes will depend increasingly on molecular biology. The genomic data of different thymoma subtypes are also essential for drug development for this disease. However, characteristic biomarkers for thymoma are insufficient compared with other tumors because thymoma is a rare group of tumors driven by a limited number of genomic events. Fortunately, several molecules have been found to be closely related to thymoma, and PI3K pathway inhibitors as a novel therapy for relapsed or refractory thymoma have been in phase II clinical trials.^11^ Our results support that high expression of KITLG is an important biomarker for type A and AB thymoma. Owing to the restriction of KITLG high expression to type A and AB thymoma, measurement of KITLG as a routine diagnostic target may be well warranted. In future, we plan to use KITLG with the MAPK pathway inhibitors to explore the possibility of the treatment level for type A and AB thymoma.

## Disclosure

The authors declare no potential conflicts of interest.
